# Paranoid Ideation in Gay Men: The Roles of Psychological Emptiness, Identity Loss, Loneliness, and Identity Resilience

**DOI:** 10.3390/healthcare14152376

**Published:** 2026-08-03

**Authors:** Barbara Lopes, Rusi Jaspal, Anthony J. Gifford

**Affiliations:** 1 The Center for Research in Neuropsychology and Cognitive and Behavioral Intervention (CINEICC), University of Coimbra, 3000-115 Coimbra, Portugal; barbaracslopes@gmail.com; 2 Vice-Chancellor’s Office, University of Brighton, Brighton BN2 4GJ, UK; 3 Department of Psychology and Counselling, Birmingham City University, Birmingham B4 7BD, UK; anthony.gifford@bcu.ac.uk

**Keywords:** psychological emptiness, identity loss, loneliness, paranoid ideation, gay men

## Abstract

**Highlights:**

**What are the main findings?**

- The cognitive-affective dispositions of psychological emptiness and identity loss are associated with feelings of loneliness, which may in turn increase the risk of paranoid ideation.
- Identity resilience may buffer the relationships between identity loss and paranoid ideation, and between identity loss and loneliness.

**What are the implications of the main findings?**

- Enhancing identity resilience in gay men who present with identity loss may reduce their risk of loneliness and paranoid ideation.
- Fostering sense of meaning and purpose, social connection, and affirmative support in those who present with psychological emptiness may also have preventive effects.

**Abstract:**

**Background/Objectives**: Gay men face mental health inequalities compared to the general population but there is limited evidence concerning the risk of paranoid ideation in this population and none that examines the cognitive-affective factors that may predispose gay men to paranoid ideation or protect against it. **Methods**: A sample of 257 gay men in the United Kingdom participated in a cross-sectional correlational survey study and completed measures of psychological emptiness, identity loss, loneliness, identity resilience, and subclinical paranoid ideation. **Results**: Hierarchical multiple regression showed that psychological emptiness was strongly associated with paranoid ideation, followed by identity loss. A moderated mediated generalized linear model showed that psychological emptiness and identity loss were associated directly with higher paranoid ideation and indirectly through the mediation of loneliness. Identity resilience moderated the relationships between identity loss and loneliness and between identity loss and paranoid ideation, essentially weakening them. **Conclusions**: Psychological emptiness and identity loss may operate as diagnostic cognitive-affective dispositions that are associated with paranoid ideation in gay men. Interventions that focus on developing a stable identity characterized by meaning and purpose may be effective against paranoid ideation and reduce the risk of severe psychopathology.

## 1. Introduction

Recent research into paranoid ideation in sexual minorities has shown that paranoid ideation is common in lesbian, gay, and bisexual (LGB) individuals and that those who present with certain cognitive-affective dispositions, such as intolerance to uncertainty, may be more prone to paranoid ideation, while those who possess adaptive self-schemas, such as identity resilience, are less likely to show paranoid ideation, even when they have dispositions that have been found to be associated with increased paranoid ideation [1]. However, there is no research into the associations between cognitive-affective dispositions of psychological emptiness and identity loss and paranoid ideation in gay men, and there is limited evidence concerning the role of identity resilience in weakening the associations between these cognitive-affective dispositions and paranoid ideation.

The cognitive-affective dispositions of psychological emptiness and identity loss are characterized by the tendency to feel detached from reality and to show feelings of “emptiness” and “fragmentation” [2,3], which have been linked to the onset of severe psychopathology, such as psychosis [2,3]. In contrast, identity resilience has been conceptualized as an adaptive self-schema, that is, “a cognitive generalization about the self derived from past experience that organizes and guides the processing of self-related information contained in the individual’s social experiences” [4]. It is characterized by positive beliefs about the self and about one’s ability to overcome difficulties [5]. Identity resilience has been found to be negatively associated with depression and anxiety in gay men [6] and paranoid ideation in LGB individuals [1].

Paranoid ideation refers to the elevated mistrust of others and is characterized by beliefs of intentional harm from others that are based on perceived or “real” social threat and defeat, e.g., experiencing discrimination or bullying [7,8,9]. People with paranoid ideation exhibit a tendency to attribute negative intentions to others, as well as hostility toward others as a safety strategy [10]. Studies show that stigmatized minority groups may be at elevated risk of paranoid ideation, due partly to long-standing exposure to stressors associated with their minority identity [11]. For instance, Rippy and Newman [12] found that perceived religious discrimination was associated with subclinical paranoia in a sample of Muslim Americans. In other words, minoritized individuals may develop a predisposition to anticipate harm from others, leading to (unfounded) beliefs of persecution even in innocuous situations [8].

As a stigmatized minority group, gay men have historically faced stressors associated with their sexuality, such as rejection, homonegativity, HIV stigma and, in many cases, internalized stigma, all of which are associated with depression [13,14]. There is also evidence that they may face additional stressors from within the gay community itself, such as rejection based on particular characteristics (e.g., ethnicity, body type, or HIV status), which has similarly been linked to increased depression and anxiety [15,16,17,18]. Incidentally, exposure to intracommunity stressors may be accentuated on mobile dating “apps,” such as Grindr [19].

Exposure to stressors may precipitate identity disturbance, such as psychological emptiness and the complete loss of identity, as well as feelings of loneliness [20,21,22,23,24]. It is known that sexual minorities exhibit significant mental health inequalities, including higher rates of depression, anxiety, and suicidal ideation compared to the general population [25,26,27,28]. Yet, there is no research focusing specifically on paranoid ideation in gay men and only limited research into sexual minorities more generally (though see [1,8]).

Accordingly, this study assesses the potential cognitive-affective dispositions that may predispose gay men to paranoid ideation, as well as the protective role of identity resilience. It should be noted that this study does not focus on the precipitants of psychological emptiness, identity loss, and identity resilience, but rather on their operation as part of a system of factors influencing paranoid ideation in a sample of gay men.

### 1.1. Paranoid Ideation

Subclinical paranoid ideation is characterized by common interpersonal fears of being talked about, laughed at, and controlled, as well as more severe persecutory delusions, such as the unfounded belief that there is a conspiracy against oneself and that one’s thoughts and actions are being controlled by others [28]. Clinical and subclinical paranoia are differentiated by degree of severity and functional impairment [29].

Subclinical paranoia is relatively prevalent in the general population. Indeed, Freeman et al. [30] found that 27% of their non-clinical British sample scored in the elevated range of Green et al.’s Paranoid Thoughts Scale. There is a paucity of research into paranoid ideation in sexual minorities, although recent studies have found the presence of paranoid ideation in LGB people [1] and that it may be more prevalent in LGB compared to heterosexual people [8].

The social defeat hypothesis [31] posits that long-term exposure to social defeat, e.g., exclusion, bullying, and discrimination, can heighten the risk of schizophrenia. Paranoid ideation in sexual minorities can be thought of as a coping mechanism that is used to deal with “real” experiences of discrimination, bullying, and social disadvantage [8]. Lopes and Jaspal [1] caution that, although paranoid ideation may constitute a coping response, persistent paranoia against the backdrop of chronic social defeat and abuse, such as social put down, rejection, discrimination and ostracism, may lead to the onset of severe psychopathology, such as psychosis [32,33,34]. Therefore, chronically stigmatized minority groups, such as gay men, may exhibit increased paranoid ideation. The cognitive-affective risk factors must be understood.

### 1.2. Cognitive-Affective Risk Factors or Loneliness and Paranoid Ideation

Psychological emptiness, a chronic transdiagnostic cognitive-affective disposition, is characterized by a persistent sense of inner void, purposelessness, detachment from reality, and feelings of numbness that influence how the individual interacts with the self and others [2]. Psychological emptiness is often attributed to adverse interpersonal experiences in childhood that make the individual feel isolated, e.g., as a “shell” presented to others [34,35]. Moreover, psychological emptiness has been found to act as a separate transdiagnostic dimension, different from loneliness and hopelessness, that predicts low remission in borderline personality disorder [2], as well as poor prognosis in schizophrenia spectrum disorders [36].

Psychological emptiness correlates strongly with psychological distress, loneliness, and dissatisfaction with life, and moderately with personality disorder traits [37]. It also correlates negatively with hope and meaning in life, and positively with suicide probability [38]. In her narrative review, Lancer ([39], p. 4) refers to a “self-reinforcing, vicious circle” of emptiness, loneliness, shame, guilt, and depression, all of which have consistently been found to precipitate and maintain paranoid ideation in clinical and non-clinical populations [7,32,40,41,42].

Indeed, as Gilbert et al. [43] have argued, individuals may develop paranoid ideation when they experience abusive, cold, and rejecting family and social environments. Consequently, the paranoid individual develops negative self-beliefs (“I am alone in this world”) and negative other-beliefs (“Others reject me”) and may detach themselves from what they perceive to be a “cold” and threatening world. These strategies operate as protective self-defenses that nevertheless make the individual feel ostracized, “empty” inside, and lonely. Therefore, it is expected that psychological emptiness will be associated positively with paranoid ideation in gay men directly and indirectly through the mediation of loneliness.

Identity loss is characterized by intense insecurity and confusion regarding one’s self-concept, values, and purpose, leading to a complete breakdown of the identity structure [44]. Identity loss may ensue from chronic experiences of devaluation, social threat, and discrimination with little recourse to coping or when the available coping strategies have failed [45]. The experience of identity loss is associated with severe psychopathology, such as depersonalization, depression, substance misuse, psychosis, and suicidal ideation [44,46,47,48].

De Vries et al. [48] have argued from a phenomenological perspective that individuals who suffer from schizophrenia have a fragmented sense of self, which can lead them to feel disconnected, disembodied, and detached from themselves and indeed the world. Identity loss may plausibly be associated with paranoia, as the paranoid individual often has a poor self-concept, harbors negative other-beliefs, and engages in disengagement and avoidance as self-protective defenses against perceived harm from others [43].

It is possible that gay men who chronically experience stressors from others may come to develop psychological emptiness and identity loss [22,49,50,51]. This may in turn increase feelings of isolation and emotional numbness, thus predisposing them to mistrust of others. It is easy to see how psychological emptiness, identity loss, and paranoid ideation may reinforce one another, creating a vicious cycle. As relatively stable cognitive-affective dispositions, psychological emptiness and identity loss may increase proneness to paranoid ideation [2]. In the long term, this may lead to the onset of psychological disorders, such as psychosis, which is characterized by detachment from reality.

Identity loss is also associated with feelings of loneliness [52], which in turn has consistently been found to predict paranoid ideation [40,53]. After all, loneliness predisposes the individual to mistrust others and to be suspicious of others’ intentions [41,54]. Therefore, it is expected that identity loss should be associated with paranoid ideation both directly and indirectly through the mediation of loneliness.

### 1.3. The Protective Effect of Identity Resilience

In identity process theory [20], identity resilience is defined as an identity that is characterized by higher combined levels of self-esteem, self-efficacy, continuity, and positive distinctiveness. These are said to constitute the four principles that underpin a positive and stable sense of identity. A person’s identity resilience develops over the life course and is shaped by many factors, including personality traits, social experiences, group memberships, and so on [20]. It is possible to enhance the constituent components of identity resilience through therapeutic intervention [55,56].

Identity resilience can be conceptualized as an adaptive self-schema that reflects the individual’s own subjective belief in their capacity to interpret and overcome challenges as they occur, as well as their self-worth and self-value [5]. As such, higher levels reflect the “capacity of the identity to resist its own invalidation, devaluation or fragmentation” Breakwell, ([20], p. 581). Indeed, identity resilience has been found to be protective against a range of psychological disorders, including anxiety and depression [21,57,58]. Lopes and Jaspal [59] found identity resilience to buffer against psychological distress in an LGB sample when they were asked to recall a negative coming out experience.

More recently, Lopes and Jaspal [1] have found identity resilience to moderate the association between uncertainty intolerance and paranoid ideation in an LGB sample, with those possessing identity resilience exhibiting less paranoia despite being intolerant of uncertainty. As an adaptive self-schema, identity resilience attenuates the relationships between dispositional vulnerabilities, such as uncertainty intolerance, and paranoid ideation. Having an identity that is resilient may facilitate and activate adaptive, effective, and sustainable coping strategies [60].

It may be possible for individuals with a disposition toward identity confusion and loss to retain a degree of identity resilience [61]. When an individual experiences acute identity confusion, this self-protective schema of identity resilience may be acting in some dimensions and areas of one’s identity (e.g., promoting self-esteem in an occupational context) but be hampered in others [1]. For example, a gay man may experience confusion and loss in relation to his sexual identity but maintain identity resilience in relation to his occupational identity (see [61]).

Gay men who present with a more vulnerable psychological profile characterized by high psychological emptiness and identity loss against a backdrop of continuous stressors may have less identity resilience overall in all areas of their identity. As such, their self-concept may be completely fragmented and “empty,” thereby increasing their vulnerability to severe psychopathology [1,36]. On this basis, it is expected that identity resilience should moderate the relationships between dispositional vulnerabilities, such as psychological emptiness and identity loss, loneliness, and paranoid ideation. In other words, gay men with identity resilience should report less loneliness and, thus, less paranoid ideation, in spite of psychological emptiness and identity loss.

### 1.4. An Identity-Based Model of Paranoid Ideation

Lopes and Jaspal [1] proposed in their identity-based cognitive model of maladaptive self-schemata and paranoid ideation in LGB samples that maladaptive self-schemata, such as uncertainty intolerance, operate as transdiagnostic cognitive-affective dispositions that increase the risk of paranoid ideation. This is attributed to inherent feelings of decreased control, as well as socio-cognitive biases of overestimating and anticipating social threat [62,63]. Moreover, the model posits that identity resilience acts as an adaptive self-schema that protects against paranoid ideation by enhancing self-worth and facilitating access to social support and perseverance against adversity [1].

Studies show the effects of uncertainty intolerance as a long-term predictor of paranoia, over and above the effects of other predictors, such as anxiety, depression, and worry [64]. However, there is a paucity of research into the potential associations between other cognitive-affective dispositions, such as psychological emptiness and identity loss, and paranoid ideation in sexual minorities. Therefore, this study examines associations between psychological emptiness and identity loss as cognitive-affective dispositions that are thought to act as psychological vulnerabilities for paranoid ideation (cognitive consequence) directly and indirectly via loneliness (negative affect). Furthermore, identity resilience is predicted to operate as an adaptive self-schema that attenuates these relationships. The following hypotheses are tested:

1. Following past research that has shown that psychological emptiness and identity loss are associated with mental health issues, such as depression, suicidal ideation, and psychosis [3,45,46,47], the first hypothesis is that psychological emptiness and identity will be positively associated with paranoid ideation.
2. Since past research has found loneliness to be strongly associated with paranoid ideation and identity loss [40,41,42], it is predicted that psychological emptiness and identity loss will also be indirectly associated with increased paranoid ideation through the mediation of higher loneliness. In other words, psychological emptiness and identity loss are expected to be associated with increased loneliness, which in turn should be associated with increased paranoid ideation.
3. Given that identity resilience has been found to weaken the associations between cognitive-affective dispositions and paranoid ideation [1], it is predicted that identity resilience should moderate the direct relationships between psychological emptiness and identity loss and paranoid ideation, as well as the mediated relationships between psychological emptiness, identity loss, loneliness, and paranoid ideation. In other words, it is expected that gay men who exhibit high identity resilience but also high psychological emptiness and identity loss should report less loneliness and paranoid ideation compared to gay men who exhibit low identity resilience.

## 2. Materials and Methods

### 2.1. Design, Procedure, and Participants

A convenience sample of 257 self-identified gay men was recruited on Prolific (https://www.prolific.com/), an online participant recruitment platform, to participate in a cross-sectional correlational survey study in April 2026. The eligibility criteria were being aged 18 years or over, male, gay, and a resident of the United Kingdom. A sample of participants who met these eligibility criteria was requested on Prolific. Participants provided demographic information, namely their age, relationship status, income, and country of residence, and completed measures of psychological emptiness, identity loss, loneliness, identity resilience, and subclinical paranoid ideation.

All participants were male, gay, aged 18 years or over, and residents in the United Kingdom. Participants were aged between 18 and 77 years (*M* = 38.46, *SD* = 11.30). There was no age limit. There were 159 (61.9%) participants who reported being single and 98 (38.1%) in a relationship. In terms of annual income, 16 (6.2%) participants reported earning less than £10,000; 28 (10.9%) between £10,000 and £19,999; 45 (17.5%) between £20,000 and £29,999; 43 (16.7%) between £30,000 and £39,999; 26 (10.1%) between £40,000 and £49,999; 26 (10.1%) between £50,000 and £59,999; and 73 (28.4%) reported earning £60,000 or more.

### 2.2. Measures

The 19-item Psychological Emptiness Scale [37] was used to measure psychological emptiness on a 5-point scale (0 = *never* to 4 = *always*). Participants were asked to indicate how often they had certain feelings over the last month. Example items of the scale are: “You felt that anything that you might do is pointless” (Nothingness) and “You were disengaged, and not really caring about anything” (Detachment). The mean of all 19 items was calculated, and a higher score indicates higher psychological emptiness. The Cronbach alpha for the total was 0.97. The factors of Nothingness and Detachment showed Cronbach alphas of 0.92 and 0.96, respectively. Psychological emptiness has been found to correlate negatively with life satisfaction and positively with loneliness [37]. In this study, psychological emptiness correlated strongly with loneliness, suggesting high validity.

The 5-item Lack of Identity Subscale of the Self-Concept and Identity Measure [3] was used to measure identity loss on a 5-point scale (1 = *strongly disagree* to 5 = *strongly agree*). The scale includes items such as “I feel empty inside, like a person without a soul” and “I no longer know who I am.” The mean of all 5 items was calculated, and a higher score indicates higher identity loss (α = 0.93). Identity loss has been found to be strongly correlated with identity disturbance [3]. Moreover, in this study, identity loss was positively correlated with identity threat (*r* = 0.65, *p* < 0.001), suggesting high validity.

The 16-item Identity Resilience Index [5] was used to measure identity resilience on a 5-point Likert scale (1 = *strongly disagree* to 5 = *strongly agree*). The scale includes items such as “All in all, I am inclined to feel that I am a failure” (reverse-scored) (Self-esteem); “I am confident that I could deal efficiently with unexpected events” (Self-efficacy); “My past and present flow seamlessly together” (Continuity); and “I feel that some of my characteristics are unique to me” (Distinctiveness). The mean of all 16 items was calculated, and a higher mean score indicates higher identity resilience. The Cronbach alpha for the total was 0.87. The factors of Self-esteem, Self-efficacy, Continuity and Distinctiveness presented Cronbach alphas of 0.85, 0.81, 0.89, and 0.79, respectively. Identity resilience was negatively correlated with identity loss (*r* = −0.46, *p* < 0.001), showing its validity.

The 10-item UCLA Loneliness Scale [65] was used to measure loneliness on a 4-point scale (1 = *never* to 4 = *always*). The scale includes items such as “How often do you feel as if nobody understands you?” and “How often do you feel starved of company?”. The mean of all 10 items was calculated, and a higher score indicates higher loneliness (α = 0.94).

The 18-item Paranoia Checklist [66] was used to measure subclinical paranoid ideation on a 5-point scale (1 = *not at all applicable* to 5 = *extremely applicable*). The scale includes items such as “I need to be on my guard against others” and “I can detect coded messages about me in the press/TV/radio.” The mean of all 18 items was calculated, and a higher score indicates higher paranoid ideation (α = 0.96). Past research has shown strong reliability estimates for the paranoid checklist and its state adaptations [30,66], including in LGB samples [1,8].

### 2.3. Statistical Analyses

A post-hoc G* power analysis was conducted [67] to evaluate statistical power with 5 predictors; the alpha was set at <0.05; *N* = 257; and the assumed effect size was 0.9. Results showed a power of 0.95 for testing the hypotheses in a sample of 257 participants. The nominal value was adjusted to 0.34 to take into account the number of hypotheses tested and the correlations between variables. This suggests that we can safely accept the study’s hypotheses and avoid Type II errors [68].

Analyses were conducted in jamovi. Spearman Rho’s correlations were conducted to analyze relationships between psychological emptiness, loneliness, identity resilience, identity loss, age, relationship status (0 = *single*; 1 = *in a relationship*), income (1 = *low income*; 6 = *high income*) and subclinical paranoid ideation.

Multiple hierarchical regression analyses bootstrapped at 10,000 samples for statistical power were conducted to analyze the independent effects of cognitive-affective dispositions of psychological emptiness and identity loss upon the variance in paranoid ideation, independently of age (covariate).

A moderated mediated advanced Generalized Linear Model (GLM) [69] was performed in jamovi to analyze the independent effects of the cognitive-affective dispositions of psychological emptiness and identity loss upon the variance in paranoid ideation both directly and indirectly through the mediation of loneliness. The GLM also assessed the moderator effects of identity resilience on the associations between the cognitive-affective dispositions of psychological emptiness and identity loss, loneliness, and paranoid ideation, independently of age.

GLM was chosen as a statistical analysis because it accommodates non-normally distributed ordinal data [69]. An advantage of GLM in jamovi is that it automatically centers the predictors and the moderators, which reduces multicollinearity and makes the main effects (lower-order) terms easier to interpret. Before testing the models, we also tested for multicollinearity (variance inflation factor < 4 and tolerance > 0.25), independence, and homoscedasticity, and all assumptions were met. Additionally, Sobel tests were performed to test the statistical significance of the mediations.

## 3. Results

### 3.1. Tests of Normality

Kolmogov–Smirnov tests were conducted to test for normal distributions. Results showed that paranoid ideation [*D*(257) = 0.20, *p* < 0.001], psychological emptiness [*D*(257) = 0.13, *p* < 0.001], identity loss [*D*(257) = 0.16, *p* < 0.001], and loneliness [*D*(257) = 0.07, *p* = 0.011] were all non-normally distributed and, therefore, non-parametric tests were used.

### 3.2. Descriptive Statistics

Table 1 presents the descriptive statistics.

### 3.3. Correlations

Table 2 presents the correlations between the variables. Since age was statistically significantly associated with paranoid ideation, it was included as a covariate in the models.

### 3.4. Hierarchical Multiple Regressions

Table 3 provides an overview of the hierarchical multiple regression models predicting the variance in paranoid ideation. In Model 1, psychological emptiness was inserted as a predictor and was statistically significant, explaining 56% of the variance in paranoid ideation. In Model 2, identity loss was inserted as a predictor and was statistically significant, explaining an additional 17% of the variance in paranoid ideation. Psychological emptiness remained the strongest predictor. In Model 3, age was inserted as a predictor but was not statistically significant. Psychological emptiness and identity loss remained statistically significant predictors of the variance in paranoid ideation, supporting Hypothesis 1.

### 3.5. Advanced Moderated Mediated Generalized Linear Model

**Figure 1:**
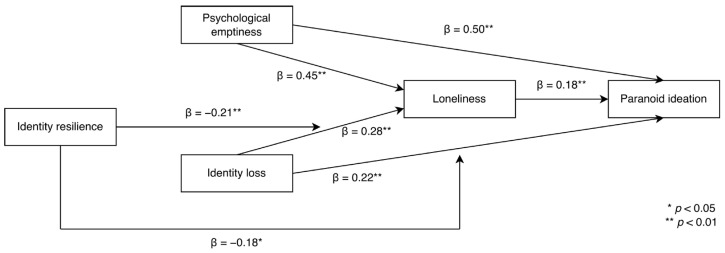
Moderated mediated model generalized linear model depicting associations between the cognitive-affective dispositions of psychological emptiness and identity loss and paranoid ideation with the moderation of identity resilience.

An advanced moderated mediated Generalized Linear Model (GLM) was constructed in jamovi (Figure 1). Psychological emptiness and identity loss were inserted as predictors; loneliness as a mediator; and paranoid ideation as the dependent variable. Identity resilience was inserted as a moderator, and age as a covariate. Table 4 presents the results of the model.

Psychological emptiness and identity loss were associated directly and positively with paranoid ideation, independently of age, which was not associated with paranoid ideation. Thus, Hypothesis 1 was supported.

Sobel tests showed that the mediated pathways between psychological emptiness, loneliness, and paranoid ideation and between identity loss, loneliness and paranoid ideation were statistically significant. Identity loss and psychological emptiness were associated positively with loneliness, which in turn was associated positively with paranoid ideation. Hypothesis 2 was supported.

There were some moderation effects. Identity resilience statistically significantly moderated the associations between identity loss and paranoid ideation, and between identity loss and loneliness. This means that gay men with high identity resilience reported less loneliness and less paranoid ideation in spite of identity loss, compared to gay men with low identity resilience. However, identity resilience did not moderate the association between loneliness and paranoid ideation, or the associations between psychological emptiness and paranoid ideation or between psychological emptiness and loneliness. Thus, Hypothesis 3 was partially supported.

## 4. Discussion

This study set out to test whether the cognitive-affective dispositions of psychological emptiness and identity loss are associated with subclinical paranoid ideation in gay men directly and indirectly through the mediation of loneliness. Moreover, the study assessed the moderation role of identity resilience upon the direct and indirect associations between psychological emptiness, identity loss, loneliness, and paranoid ideation.

As cognitive-affective dispositions, both psychological emptiness and identity loss are strongly associated with paranoid ideation, with psychological emptiness explaining a greater portion of the variance in paranoid ideation. These results suggest that the perceptual biases of psychological emptiness, which make the individual feel a void and detachment from reality, may play an important role in the development and maintenance of paranoid ideation [36]. After all, the paranoid individual often feels detached and isolated from the world and engages in disengagement and avoidance of others as self-protective strategies [43].

Gay men may be at particularly high risk of psychological emptiness. In their study of 366 gay and bisexual men, He et al. [49] found that almost 50% of participants exhibited medium risk of psychological emptiness and self-criticism and almost 10% exhibited high risk. In another study, He et al. [49] found that higher use of a gay dating app, itself very common in gay men, was associated with higher psychological emptiness. Moreover, gay men’s narratives of engaging in “chemsex” (drug use in sexualized settings) tend to feature the theme of psychological emptiness—gay men may attempt to fill an emotional void by engaging in substance use in sexualized settings (e.g., [70,71,72]).

Identity loss may similarly operate as a critical cognitive-affective disposition that makes the individual feel vulnerable, lost, and devoid of meaning and purpose [3]. These feelings may make the individual feel scrutinized, alone, and mistrustful of others [66]. Indeed, the lack of a stable self-concept that is observed in identity loss may be attributed to negative social experiences. For instance, gay men report being rejected and ostracized due to their sexuality, which may prompt identity confusion and, in some cases, loss [50]. This in turn may increase paranoid ideation as a means of coping with prolonged abuse and threats to identity [1,8].

In addition to their direct associations with paranoid ideation, both psychological emptiness and identity loss were associated indirectly with paranoid ideation through the mediation of increased loneliness. Indeed, both psychological emptiness and identity loss may make the individual feel “estranged” from the world and, as such, lonely and lacking in social support and meaningful connections. These have been found to be strong predictors of paranoid ideation [40,41].

Gay men appear to be at high risk of experiencing loneliness due primarily to exposure to stressors in their social environment, which in turn may be internalized at the psychological level. In their systematic review and meta-synthesis, Brumfield and Dahlenburg [73] identify three clusters of factors that influence gay men’s experiences of loneliness: external influences of discrimination; internal conflicts; and choice of coping strategy (see also [74]). In addition to noting the effects of homonegativity, Kromholz and Barak [23] describe some of the intracommunity dynamics (e.g., behaviors on gay dating apps, casual sexual encounters) that contribute to feelings of loneliness in gay men.

In support of Lopes and Jaspal’s [1] identity-based cognitive model of maladaptive self-schemata and paranoid ideation in LGB individuals, our results show that identity resilience moderated the direct association between identity loss as a cognitive-affective vulnerability and paranoid ideation (cognitive consequence), as well as the association between identity loss and loneliness (negative affect). This suggests that gay men with high identity resilience report less loneliness and may therefore show less paranoid ideation, despite possessing the cognitive-affective predisposition of identity loss.

Identity loss refers to the breakdown of the identity structure [3]. Therefore, it could be argued that a person experiencing identity loss, by definition, cannot have an identity that is resilient. However, the onset of identity loss is often acute, arising in response to a traumatic event or a series of cumulative stressors [50,51]. The individual experiencing identity loss may nonetheless retain remnants of self-esteem, self-efficacy, continuity, and positive distinctiveness in a general sense. After all, identity consists of multiple elements, and these psychological needs may be satisfied by other elements of identity. For instance, a gay man may experience stressors relating to his sexuality but derive self-esteem based upon other elements of his identity. Our findings suggest that possessing higher identity resilience may offer some protection against the harmful effects of identity loss. It is possible that identity resilience facilitates access to adaptive coping strategies, such as the derivation of social support, which may prevent feelings of loneliness and, as such, protect against the development and maintenance of paranoid ideation [1].

It should be noted that identity resilience moderated neither the direct association between psychological emptiness and paranoid ideation nor their indirect association through loneliness. Furthermore, identity resilience did not moderate the association between loneliness and paranoid ideation. It appears to offer no protection against the cognitive-affective disposition of psychological emptiness or against the negative affective state of loneliness.

Since they feel detached from the world, gay men with psychological emptiness appear to be at high risk of developing loneliness regardless of the level of identity resilience. Furthermore, their sense of isolation and detachment from others may make gay men who are lonely more susceptible to paranoid ideation regardless of identity resilience. Identity resilience essentially reflects the individual’s appraisal of their own identity. This may be insufficient to reduce the effects of psychological emptiness on loneliness and paranoid ideation, showing the limits of identity resilience (cf. [20]).

### 4.1. Clinical Implications

Psychological support that focuses on developing a stable sense of self, meaning and purpose may be effective in alleviating psychological emptiness, identity loss, and paranoid ideation in gay men. From a clinical perspective, psychological emptiness and identity loss should be considered cognitive-affective dispositional vulnerabilities that are characterized by a lack of purpose, goals, and meaning, which in turn create an existential vacuum [75]. Psychological emptiness and identity loss are associated with a history of exposure to stressors, such as discrimination.

It is important to build a clinical psychological profile of stigmatized and vulnerable gay men who may present chronic cognitive-affective dispositions of psychological emptiness and identity loss that consist of symptoms of nothingness, confusion, fragmentation, and isolation from the world [76]. Moreover, mental health professionals must recognize that paranoid ideation in stigmatized groups, such as gay men, may constitute a coping response to stressors. However, in the long term, gay men who present with the cognitive-affective dispositions of psychological emptiness and identity loss may be vulnerable to the development of severe mental health disorders [32].

Psychotherapeutic interventions must address the cognitive-affective dispositions of psychological emptiness and identity loss and encourage the pursuit of meaning and of identity re-construction while fostering social connection and affirmative support [77]. Mindfulness has been found to be effective in promoting psychological meaning and engagement by focusing the individual on the present moment and on engaging with the world [78]. Moreover, identity-reframing interventions appear to help individuals develop a stable and positive identity by promoting feelings of self-worth, finding purpose, and focusing on individual strengths [79]. A sexuality-affirmative approach to psychotherapeutic intervention will be key.

### 4.2. Limitations

It is not possible to infer causation based on this cross-sectional correlational survey study. Future studies should recruit larger samples of gay men and other sexual minorities and longitudinally test the relations between psychological emptiness, identity loss, loneliness, and paranoid ideation. Future research should also experimentally test whether identity resilience attenuates the associations between psychological emptiness, identity loss, and paranoid ideation. Moreover, this study focused exclusively on gay men. Studies should test the model in other sexual minorities, such as lesbian women and bisexual individuals. The study did not measure stressors, such as discrimination and internalized homophobia, that may precipitate psychological emptiness and identity loss and prevent access to social support. These may be important confounding factors that should be measured in the future. In addition, the study did not measure education level, regional identity (e.g., rural vs. urban), or ethnicity, all of which may have had an impact on the results. This limits the generalizability of the results.

## 5. Conclusions

Gay men may develop cognitive-affective dispositions of psychological emptiness and identity loss due to long-term exposure to stressors, which in turn may be associated with increased loneliness and paranoid ideation. Identity resilience may act as a protective self-schema that attenuates the relationship between identity loss and increased loneliness and, thus, paranoid ideation. Therefore, fostering identity resilience and promoting the search for meaning and purpose in gay men who are vulnerable to severe psychopathology due to the emotional numbness, detachment of reality, and fragmentation commonly observed in identity loss is important.

## Figures and Tables

**Table 1:** Descriptive statistics.

Variables	*M*	*SD*	Min	Max	Skewness	Kurtosis
Age	38.5	11.3	18	77	0.72	0.01
Psychological Emptiness	1.83	0.74	1	4	0.77	−0.38
Identity Loss	2.10	1.04	1	5	0.82	−0.15
Loneliness	2.32	0.75	1	4	−0.00	−0.75
Identity Resilience	3.50	0.60	1.38	5	−0.30	0.30
Paranoid Ideation	1.84	0.84	1	4.33	1.04	0.03

**Table 2:** Correlations.

Variables	1	2	3	4	5	6	7
1. Age							
2. Relationship status	0.15 *						
3. Income	0.23 **	0.17 **					
4. Psychological emptiness	−0.23 **	−0.02	−0.21 **				
5. Identity loss	−0.18 **	−0.08	−0.10	0.61 **			
6. Loneliness	−0.09	−0.16 **	−0.14 *	0.64 **	0.56 **		
7. Identity resilience	0.12 *	0.12	0.39 **	−0.51 **	−0.43 **	−0.44 **	
8. Paranoid ideation	−0.16 *	0.02	−0.11	0.57 **	0.50 **	0.50 **	−0.30 **

** *p* < 0.01 * *p* < 0.05.

**Table 3:** Hierarchical multiple regressions predicting paranoid ideation.

	*R* ^2^	*F*	*p*	β	*t*	*p*	95% CI
**Model 1**	0.31	116.78	**<0.001**				
Psychological emptiness				0.56	10.80	**<0.001**	0.52, 0.75
**Model 2**	0.33	62.428	**<0.001**				
Psychological emptiness				0.45	6.67	**<0.001**	0.36, 0.66
Identity loss				0.17	2.43	**0.016**	0.003, 0.24
**Model 3**	0.33	42.35	**<0.001**				
Psychological emptiness				0.44	6.48	**<0.001**	0.35, 0.65
Identity loss				0.16	2.35	**<0.019**	0.02, 0.24
Age				−0.07	−1.34	0.18	−0.01, 0.00

**Table 4:** Moderated mediation model predicting paranoid ideation.

	β	*Z*	*S.E.*	*p*	95% CI	Sobel Test *Z*	*p*
**Direct effects**							
Psychological emptiness > paranoid ideation	0.50	6.90	0.08	**<0.001**	0.40, 0.72		
Identity loss > paranoid ideation	0.22	3.12	0.06	**0.002**	0.07, 0.28		
Loneliness > paranoid ideation	0.18	2.68	0.08	**0.007**	0.05, 0.35		
Age > paranoid ideation	−0.04	−0.82	0.00	0.41	−0.01, 0.00		
Identity resilience > paranoid ideation	−0.26	−4.41	0.08	**<0.001**	−0.39, −0.15		
Psychological emptiness > loneliness	0.45	7.03	0.07	**<0.001**	0.33, 0.59		
Identity loss > loneliness	0.28	4.42	0.08	**<0.001**	0.05, 0.35		
Age > loneliness	0.09	1.99	0.00	**0.046**	1.10, 0.01		
**Moderation effects**							
Psychological emptiness × identity resilience > paranoia	−0.06	−0.61	0.15	0.54	−0.38, 0.20		
Identity loss × identity resilience > paranoid ideation	−0.18	−2.09	0.09	**0.037**	0.01, 0.38		
Age × identity resilience > paranoid ideation	0.00	0.15	0.00	0.88	−0.01, 0.01		
Loneliness × identity resilience > paranoid ideation	−0.12	−0.62	0.11	0.54	−0.27, 0.34		
Psychological emptiness × identity resilience > loneliness	−0.04	−0.48	0.12	0.63	−0.28, 0.17		
Identity loss × identity resilience > loneliness	−0.21	−2.66	0.08	**0.008**	−0.05, −0.35		
Age × identity resilience > loneliness	0.02	−0.49	0.00	0.62	−0.00, 0.001		
**Mediations**							
Psychological emptiness > loneliness > paranoid ideation	0.08	2.50	0.04	**0.012**	0.02, 0.17	7.40	**<0.001**
Identity loss > loneliness > paranoid ideation	0.05	2.29	0.02	**0.022**	0.00, 0.08	6.69	**<0.001**
Age > loneliness > paranoid ideation	0.02	1.60	7.92	0.11	−2.85, 0.00	0.00	n.s.

## Data Availability

The data are openly accessible on OSF: https://doi.org/10.17605/OSF.IO/8QD7T.
